# Motivation to quit smoking among HIV-positive smokers in Vietnam

**DOI:** 10.1186/s12889-015-1672-y

**Published:** 2015-04-03

**Authors:** Nhung Thi Phuong Nguyen, Bach Xuan Tran, Lu Y Hwang, Christine M Markham, Michael D Swartz, Jennifer I Vidrine, Huong Thu Thi Phan, Carl A Latkin, Damon J Vidrine

**Affiliations:** Department of Pharmacoeconomics & Pharmacoepidemiology, Hanoi University of Pharmacy, 13-15 Le Thanh Tong St, Hanoi, Vietnam; Institute for Preventive Medicine and Public Health, Hanoi Medical University, Hanoi, Viet Nam; Department of Health, Behavior and Society, Johns Hopkins Bloomberg School of Public Health, Baltimore, MD USA; The University of Texas Health Science Center at Houston, Houston, TX 77030 USA; Department of Health Disparities Research, Division of OVP, Cancer Prevention and Population Sciences, The University of Texas MD Anderson Cancer Center, Houston, TX USA; Department of Behavioral Science, The University of Texas MD Anderson Cancer Center, Unit 1330, Houston, TX 77030 USA; Authority of HIV/AIDS Control, Ministry of Health, Hanoi, Vietnam

**Keywords:** Cigarette, Smoking, Quit, Cessation, HIV, Vietnam

## Abstract

**Background:**

Smoking cessation is emerging as an important component in current HIV care to reduce smoking-related adverse health outcomes. This study aimed to examine motivation to quit and its associated factors in a sample of 409 HIV-positive smokers in Vietnam.

**Methods:**

A cross-sectional survey was conducted from January to September 2013 in Hanoi (the capital) and Nam Dinh (a rural city). Motivation to quit was measured by a 4-point single item, and was dichotomized as having any motivation versus no motivation. Smoking history, nicotine dependence (Fagerstrom Test of Nicotine Dependence), and other covariates were self-reported by participants. Multivariate logistic regression was performed to identify correlates of motivation to quit.

**Results:**

The sample was mostly male (97%). Mean age was 36 years (SD = 5.8). Approximately 37% and 69% of the sample were hazardous drinkers and ever drug users, respectively. The mean duration of HIV infection and ART treatment were 6 years (SD = 3.6) and 5 years (SD = 2.2), respectively. Overall, 59% of the sample was motivated to quit. Factors significantly associated with motivation to quit were income, pain, currently taking Methadone, and the interaction between binge drinking and lifetime drug use. Individuals with the highest income level (OR = 2.2, 95% CI = 1.3-3.6), moderate income level (OR = 1.8, 95% CI = 1.1-3.1), and currently feeling pain (OR = 1.6, 95% CI = 1.0-2.5) were more likely to be motivated to quit. Conversely, taking Methadone was associated with a lower likelihood of motivation to quit (OR = 0.4, 95% CI = 0.2-0.9). Also, those who reported binge drinking only (OR = 0.5, 95% CI = 0.3-0.9), lifetime drug use only (OR = 0.3, 95% CI = 0.1, 0.7), or both substance uses (OR = 0.4, 95% CI = 0.2, 0.8) were less motivated to quit smoking.

**Conclusion:**

Smoking cessation treatment should be integrated into HIV care in Vietnam, and should be tailored to meet specific needs for individuals with different attitudes on smoking, low income, and polysubstance use.

## Background

Cigarette smoking among people living with HIV/AIDS (PLWHA) represents a growing public health problem [1,2]. The combination of high smoking prevalence and prolonged survival among PLWHA places this vulnerable group at an elevated risk of chronic smoking-related conditions (e.g., cardiovascular diseases, non-AIDS cancers) [1]. Therefore, efficacious smoking cessation treatment offers the potential to reduce morbidity and mortality for PLWHA. Research indicates that smoking cessation among HIV-positive smokers could decrease the risk of overall mortality by 16%, the risk of cardiovascular diseases by 20%, and the risk of non-AIDS malignancies by 34% [3]. Also, an increased time length of smoking abstinence is associated with decreased HIV-related symptom burden [4] and decreased risk of cardiovascular diseases [5]. Collectively, smoking cessation is emerging as an important component in current HIV care [6].

Available data indicate a mixed level of desire to quit smoking among HIV-positive smokers, with approximately 37-75% thinking about quitting [7-11]. While many HIV-positive smokers are interested in quitting and have made quit attempts, their success rates are low [2]. Gritz et al. reported that the abstinence rate among PLWHA was nearly half the estimate for the general population [8]. The overlap between HIV/AIDS, low socioeconomic status, substance abuse, and mental illnesses may make smoking abstinence more difficult among PLWHA as compared to the general population [9,12]. To help PLWHA quit successfully, predictors of smoking cessation should be identified, and efforts to match HIV-positive smokers with appropriate interventions should be evaluated. According to theories of health behavior change (e.g., the Theory of Planned Behavior [13], the Transtheoretical Model [14]), motivation to quit directly increases intention to quit, facilitates cessation attempts, and increases successful abstinence rates. However, motivated smokers may not have the intention to quit due to barriers related to quitting. Thus, cessation interventions are needed to increase readiness to quit smoking and to promote abstinence [15]. To design effective cessation interventions, a thorough understanding of motivation to quit is critical to explore facilitators and barriers related to quitting, and to elucidate teachable moments. To date, there is a paucity of research on motivation to quit among HIV-positive smokers, especially in developing countries, where both smoking and HIV epidemics have severe effects [16].

Viet Nam is a developing country confronting detrimental impacts from the intersection of cigarette smoking and HIV/AIDS. The estimates of current cigarette smoking in the general population were approximately 19.9%, higher in males (39.7%) than in females (1.2%) [17]. Furthermore, smoking is considered as an accepted behavior among men and accounts for 28% of all adult male deaths [18]. The cost of three smoking-related diseases (i.e., lung cancer, heart disease, and chronic obstructive pulmonary disease) was 77.5 million USD, approximately 4.3% of the total healthcare expenditure [19]. In addition to this smoking epidemic, Viet Nam remains in a concentrated HIV epidemic, which is mainly driven by drug users. The HIV prevalence among adults ages 15–49 years is 0.45% with 282,787 PLWHA nationwide [20]. Vietnam is ranked fifth among twelve countries with highest HIV burden in Asian and Pacific region [21]. Of note, our exploratory study observed a high smoking rate (36.1%) and a low lifetime cessation rate (i.e., 20.9% of ever smokers have successfully quit) in a sample of 1133 PLWHA in Vietnam [22] . Additionally, previous studies revealed that approximately 70% of PLWHA use illicit drugs [23] and 55% use alcohol [24] in Vietnam. Collectively, engaging in other substances and cultural perspectives may be unique barriers for quitting smoking among HIV-positive smokers in this developing country. To our knowledge, there has been no study about motivation to quit among PLWHA in Vietnam. Therefore, this study aimed to examine motivation to quit and its correlates in a sample of HIV-positive smokers. Findings from the study may add to limited evidence on this issue in general, and inform future cessation interventions targeting to HIV-positive smokers in Vietnam in particular.

## Methods

### Study design and settings

A cross-sectional survey was conducted in Hanoi (the capital) and Nam Dinh (a rural city) from January to September 2013. These two cities are among the areas with the largest HIV infection burden in the northern region in Vietnam, with 20,762 and 3781 PLWHA in Hanoi and Nam Dinh, respectively [20]. Five antiretroviral therapy (ART) clinics in Hanoi and three ART clinics in Nam Dinh were purposively selected based on the following criteria: 1) including central, provincial, and district level sites; 2) providing ART services; and 3) having a sufficient number of HIV/AIDS patients.

### Study subjects

Patients were approached and recruited through a convenience sampling technique. To be eligible, patients must have been 18 years or older, HIV-positive, currently taking ART, and willing to provide written informed consent. Individuals having any serious health problems that could interfere with participating in an interview were excluded from the study. All eligible patients presenting at the clinics during the study period were identified and invited to participate in the survey until a sufficient sample size of at least 100 patients per provincial or district site, and 200 patients per central site, was obtained. Participants were asked to complete a 30–45 minute face-to-face interview.

### Measures

#### Outcome variable

The primary outcome was motivation to quit smoking. Based on the Contemplation Ladder [15], a common measure of readiness to quit smoking, we devised a single item “Currently, what is your thinking about quitting smoking?” to measure motivation to quit. Responses included “No thought of quitting”, “Think I should quit but not quite ready”, “Starting to think about how to change my smoking patterns”, and “Take action to quit". The outcome was dichotomized in data analyses. That is, participants who chose “No thought of quitting” were considered as having no motivation to quit. In contrast, those who chose the remaining responses were categorized as having motivation to quit. Additionally, motivated participants were asked when they planned to quit smoking from the time of interview, and what cessation supports they preferred.

#### Covariate variables

Smoking-related characteristics: Based on validated smoking instruments used in previous studies [25,26], we also devised items to assess other smoking indicators (e.g., age at smoking initiation, number of cigarettes smoked per day). We employed the Fagerstrom Test for Nicotine Dependence (FTND), a well-validated instrument, to evaluate the level of nicotine dependence [27]. This measure included six items, yielding a total score of 0–10 with higher scores indicating higher nicotine dependence [27].

Sociodemographic characteristics (i.e., age, gender, educational attainment, marital status, income, and employment status) were obtained. The annual income was calculated by summing up all sources of income during the last 12 month, and then stratified into three quantiles.

Other risk behaviors were queried, including alcohol and illicit drug use. Alcohol consumption was measured by the brief version of the Alcohol Use Disorders Identification Test-Consumption (AUDIT-C), a screen for heavy drinking and/or active alcohol abuse at healthcare clinics [28]. This instrument includes three questions: 1) “How often do you have a drink containing alcohol?”; 2) “How many standard drinks containing alcohol do you have on a typical day?”; and 3) “How often do you have six or more drinks on one occasion?”. The response score for each item ranged from 0 to 4, making the total score scale range from 0 to 12. Higher scores indicated the higher likelihood patients were at risk of alcohol dependence. Threshold scores of 4 or above in men and 3 or above in women were used to identify hazardous drinkers. In addition, binge drinking is defined by any positive response to the third question. The Vietnamese version of AUDIT-C instrument has been validated in previous studies [24,29]. Illicit drug use was examined in terms of history of drug use, current drug use, duration of drug use, type of drugs, and route of drug use [24]. Health-related characteristics were self-reported, and included weight, height, HIV-infection duration, current HIV stage, recent CD4 cell count, and ART treatment duration. CD4 cell count was defined as the number of CD4 cells per cubic millimeter (mm^3^) of blood; this was dichotomized at the cut point of 200 cells per mm^3^ of blood. Feeling pain and anxiety were assessed by two items from the EQ-5D-5 L, a measure of health-related quality of life for PLWHA [30]. Accordingly, patients who answered “Slightly” to “Extremely” were considered as currently having pain or anxiety. The Vietnamese version of EQ-5D-5 L instrument has been validated in a previous study [31].

### Data analyses

Descriptive statistics were conducted to compare sociodemographic characteristics, substance use behaviors, and health-related characteristics by level of motivation to quit (Having motivation to quit vs. No motivation to quit). Student´s t-test and Mann–Whitney U-test were computed to examine differences between means of normally and non-normally distributed variables, respectively. Chi-square tests were used for categorical variables. Frequencies and distributions of smoking-related items by level of motivation to quit were also described. Multiple logistic regression modeling was performed to identify factors significantly associated with motivation to quit. Models were built based on the strategy recommended by Hosmer & Lemeshow [32]. Accordingly, variables whose univariate tests had a p-value <0.25 were candidates for the multivariate model (i.e., income, marital status, employment status, CD4 cell count, feeling pain and anxiety, currently taking Methadone) along with all variables of known clinical importance (i.e., binge drinking, ever used drugs). We then applied a stepwise backward model approach based on the log-likelihood ratio test including variables with a p-value < 0.1. Collinearity was checked by variance inflation factors with a cut off value of 10. All potential interactions were examined. We assessed model calibration using Hosmer-Lemeshow goodness-of-fit test [32]. All tests of hypotheses were two-tailed with a significance level of α less than 0.05. Statistical analyses were performed with STATA version 12.0 (StataCorp LP, College Station, Texas, USA).

### Ethical issues

The use of data for this analysis was approved by the Vietnam Authority of HIV/AIDS Control's Scientific Research Committee and the Institutional Review Board at The University of Texas Health Science Center at Houston.

## Results

### Sample characteristics

In total, 1258 patients were identified and invited to participate in the survey. Of these, 1133 PLWHA were surveyed (response rate 90%) and 409 current smokers were identified and included in this data analysis. The study sample was predominately male (97%) and had a mean age of 36 years (SD = 5.8). The majority was from Hanoi (87%), lived with a spouse/partner (67%), had less than a high school education (56%), and was currently working (76%). The mean AUDIT-C score was 2.9 (SD = 2.9). Approximately 37% and 43% of the sample were hazardous drinkers and binge drinkers, respectively. More than two thirds of the sample (69%) reported ever using illicit drugs. Nevertheless, only 5 % were current drug users, and 6% were in Methadone maintenance treatment. Most participants (75%) had a recent CD4 count of 200 or more cells/mm^3^, and 83% reported having normal weight. Approximately 14% of participants were diagnosed with AIDS. The mean duration of HIV infection and ART treatment were 6 years (SD = 3.6) and 5 years (SD = 2.2), respectively. Pain (34%) and anxiety (41%) were prevalent in the study sample (Table 1).

**Table 1:** **Characteristics of study participants (n = 409)**

**Characteristics**		**Total**	**Motivated to quit**		**p-value**
			**Yes (n = 243, 59%)**	**No (n = 166, 41%)**	
**Sociodemographics, % (n)**					
Age in years, mean (sd)		36.05 (5.8)	36.08 (5.8)	36.0 (5.7)	0.89
Gender					
Female		3 (12)	3 (6)	4 (6)	0.50
Male		97 (397)	88 (237)	96 (160)	
Annual income					
Lowest (<1.2 million VND)		34 (139)	29 (70)	42 (69)	0.01
Moderate (1.2-3.0 million VND)		35 (142)	40 (96)	28 (46)	
Highest (>3.0 million VND)		30 (124)	31 (76)	29 (48)	
Marital status					
Live with spouse/partner		67 (273)	69 (168)	63 (105)	0.22
Education					
Less than high school		56 (230)	56 (136)	57 (94)	0.64
High school		35 (143)	34 (83)	36 (60)	
More than high school		9 (36)	10 (24)	7 (12)	
Location					
Hanoi		87 (354)	86 (210)	87 (144)	0.92
Nam Dinh		13 (55)	14 (33)	13 (22)	
Employment status					
Currently working		76 (309)	81(196)	68 (113)	<0.01
**Substance use, % (n)**					
AUDIT-C score, mean (sd)		2.9 (2.9)	2.7 (2.8)	3.1 (3.0)	0.17
Hazard drinking		37 (151)	35 (85)	40 (66)	0.33
Binge drinking		43 (177)	39 (94)	50 (83)	0.02
Lifetime drug use		69 (282)	65 (159)	74 (123)	0.06
Current drug use		5 (20)	3 (8)	7 (12)	0.07
Currently taking Methadone		6 (25)	4 (10)	9 (15)	0.04
**Health-related characteristics, % (n)**					
Recent CD4 ≥ 200 cells per mm^3^		75 (236)	71 (130)	81 (106)	0.046
BMI					
Under weight (<18.5 kg/m^2^)		9 (36)	10 (24)	7 (12)	0.64
Normal weight (18.5-22.9 kg/m^2^)		83 (338)	82 (199)	84 (139)	
Over weight (> = 23 kg/m^2^)		8 (35)	8 (20)	9 (15)	
HIV stage					
Without symptom		36 (149)	50 (85)	56 (64)	0.37
With symptom		20 (80)	31 (53)	24 (27)	
AIDS		14 (56)	19 (32)	21 (24)	
HIV duration (years), mean (sd)		6.1 (3.6)	6.0 (3.5)	6.3 (3.8)	0.46
ART duration (years), mean (sd)		4.5 (2.2)	4.4 (2.1)	4.5 (2.2)	0.71
Currently feeling pain		34 (138)	37 (90)	29 (48)	0.09
Currently feeling anxiety		41 (168)	44 (106)	37 (62)	0.21

### Smoking-related characteristics

Smoking-related characteristics are presented in Table 2. The mean age of smoking initiation was 17 years (SD = 4.1), corresponding to an average duration of smoking of 13 years (SD = 8.1). The mean Fagerstrom score was 3.6 (SD = 2.1), with 34% at very low dependence, 33% at low dependence, 12% at moderate dependence, 15% at high dependence, and 5% at very high dependence. Overall, 59% of the sample was motivated to quit, and 33% had made at least one quit attempt in the past year. Among motivated smokers, 24% and 42% planned to quit within the next month and the next 1–3 months at the time of interview, respectively. Regarding types of cessation support, self-help was the most frequently endorsed method selected (39%).

**Table 2:** **Smoking-related characteristics of study participants**

**Smoking-related characteristics**	**Total**	**Motivated to quit**		**p-value**
		**Yes (n = 243)**	**No (n = 166)**	
**Age at smoking initiation,** mean (sd)	17.0 (4.1)	17.2 (3.8)	16.8 (4.4)	0.38
**Number of cigarettes per day,** % (n)				
<10	62 (250)	63 (152)	59 (98)	
11-20	35 (141)	33 (79)	37 (62)	0.84
21-30	2 (6)	2 (4)	1 (2)	
>30	1 (5)	1 (3)	1 (2)	
**Duration of regular smoking in years,** mean (sd)	13.3 (8.1)	13.2 (7.9)	12.5 (8.6)	0.79
**Smoke within 5 min of waking,** % (n)	27 (109)	25 (61)	29 (48)	0.54
**FTND score,** mean (sd)	3.6 (2.1)	3.6 (2.1)	3.8 (2.2)	0.38
**Nicotine dependence level,** % (n)				
Very low	34 (140)	34 (82)	35 (58)	
Low	33 (136)	35 (84)	31 (52)	
Moderate	12 (50)	14 (34)	10 (16)	0.40
High	15 (62)	14 (33)	18 (29)	
Very high	5 (21)	4 (10)	7 (11)	
**Quit attempts within the last 12 months,** % (n)	33 (135)	50 (122)	8 (13)	<0.01
**Plan to quit,** % (n)				
Within 30 days (1 month)		24 (58)		
1 to 3 months from now		42 (103)		
3 to 6 months from now	NA	8 (19)	NA	
6 to 12 months from now		4 (9)		
1 or more years from now		1 (3)		
Don’t know		21 (51)		
**Preferences for cessation support,** n (%)				
Health staff support		3 (7)		
Family support		6 (15)		
Friend/peer support		1 (3)		
Using Nicotine replacement therapy	NA	13 (31)	NA	
Using herbs		1 (2)		
Using acupuncture		1 (3)		
Use mobile phone		0 (1)		
Self-help		63 (154)		

### Factors associated with motivation to quit

Few differences were apparent between motivated and non-motivated HIV-positive smokers. Participants who were motivated to quit were more likely to make quit attempts in the past year (50%), had a higher income, and were currently working. Conversely, non-motivated smokers were more likely to be binge drinkers, currently taking Methadone, and had a CD4 count of 200 or more cells/mm^3^ (Table 1).

Results from the multivariate logistic regression are presented in Table 3. In the univariate analyses, factors significantly related to motivation to quit were income, employment status, binge drinking, CD4 cell count, and the interaction between binge drinking and lifetime drug use (data not shown). After adjusting for other covariates, those retained in the multiple regression model were income, the interaction between binge drinking and lifetime drug use, taking Methadone, and pain. Compared to smokers with the lowest income level, those with the highest level (OR = 2.2, 95% CI = 1.3-3.6) and the moderate level (OR = 1.8, 95% CI = 1.1-3.1) had a higher likelihood of being motivated to quit. Similarly, individuals who currently had pain (OR = 1.6, 95% CI = 1.0-2.5) were more likely to be motivated to quit. Lifetime drug use alone was not a significant predictor. Rather this factor interacted with alcohol consumption to reduce the likelihood of quit motivation. Accordingly, smokers who reported binge drinking only (OR = 0.5, 95% CI = 0.3-0.9), lifetime drug use only (OR = 0.3, 95% CI = 0.1, 0.7), or both substance uses (OR = 0.4, 95% CI = 0.2, 0.8) were less motivated to quit compared to those who never used drugs and did not binge drink. Of interest, participants who were currently taking Methadone were less likely to be motivated to quit, compared to those who were not taking Methadone (OR = 0.4, 95% CI = 0.2, 0.9) (Table 3).

**Table 3:** **Associated factors of motivation to quit**

	**Multivariate regression**	
	**Adjusted OR (95% CI)**	**p-value**
**Sociodemographics**		
Income		
Lowest	1.00	
Moderate	2.20 (1.33-3.62)	<0.01
Highest	1.84 (1.09-3.10)	0.02
**Substance use**		
Interaction between alcohol and drug use		
*Never use drugs + No binge drinking*	1.00	
*Binge drinking only*	0.47 (0.25-0.89)	0.02
*Lifetime drug use only*	0.31 (0.14-0.67)	<0.01
*Lifetime drug use + Binge drinking*	0.40 (0.20-0.77)	<0.01
Currently taking Methadone (Yes vs. No)	0.40 (0.17-0.94)	0.04
**Health-related characteristics**		
Currently feeling pain (Yes vs. No)	1.60 (1.02-2.52)	0.04

## Discussion

To our knowledge, the current study is among the first to address motivation to quit smoking cigarettes among PLWHA in Vietnam. Results revealed that a substantial proportion of HIV-positive smokers were motivated to quit (59.4%) in this developing country. Although the estimate was lower than that in the Vietnamese general population (67.5%) [17], it was in an upper bound of a range reported among HIV-positive cohorts worldwide [7-10,33,34].

While our motivation to quit rate is encouraging, we observed a large discrepancy in attempting to quit by level of quit motivation. That is, among motivated individuals, more than half (50.2%) had made at least one quit attempt in the past year and two thirds (66.3%) planned to quit in the next 3 months. On the other hand, very few non-motivated smokers (7.8%) reported attempting to quit in the past year. These findings are congruent with the conceptual framework proposed by Vidrine et al. [35] in which smoking attitudes influence changes in smoking behavior. Also, previous research indicates that motivation to quit is positively associated with a number of quit attempts [36,37] and successful abstinence [11,38]. Therefore, motivated smokers are the best candidates for cessation interventions, and should be targeted with evidence-based treatments to increase quit rates [39,40]. For those not interested in quitting, motivational enhancement and/or psychoeducational approaches may be needed to change beliefs and attitudes related to smoking, and to increase quit motivation before providing more traditional cessation treatment [40]. Taken together, cessation treatment efficacy could be improved through tailoring based on an individual’s attitudes about smoking, including level of quit motivation.

This study extends the evidence base in the literature by identifying facilitators and barriers for motivation to quit among HIV-positive smokers in a developing country. We found that higher income and pain were positively associated with quit motivation. This is consistent with earlier reports that have identified economical disadvantages (i.e., low income) as predictors of poor cessation outcomes among HIV-positive smokers [41,42]. It is likely that participants with low income have limited resources for, and limited access to cessation treatment, compared to higher income individuals [40]. Even the average annual income per capita of our sample (US$132/year) was much lower than that in the Vietnamese general population (US$1730) [43], the socioeconomics disadvantages continue to present among this low-income group. Therefore, providing free cessation treatment may encourage quitting smoking in this underserved group.

Additionally, we observed that HIV-positive smokers currently having pain were more likely to be motivated to quit. There is inconsistent evidence regarding the smoking–pain relationship among PLWHA. Some research suggests that smoking may help PLWHA to cope with pain [36,44]. Therefore, pain may be a barrier to quitting. In contrast, pain could be a facilitator for smoking abstinence. Other research, such as Vidrine et al.’s framework [35], indicates that smokers with higher perceived impact of an HIV-related event (i.e., pain, or symptom burden) may be more likely to change their attitudes on smoking. Future research should fully elucidate this association.

Variables associated with being unmotivated to quit smoking included codependence with other substances. In line with prior research [36,39,41], we did observe that HIV-positive smokers with alcohol and/or drug use were less motivated to quit. These major barriers for smoking cessation are not especially unique to HIV-positive populations, but rather also observed among other populations [44]. A possible explanation is that smoking and other substance use may be reciprocally reinforced behaviors due to the shared neurobiological mechanisms (i.e., common genetic factors, the dopaminergic neurotransmission) [45] and the similar psychological and physical cues and withdrawal symptoms (e.g., craving, stress) [46]. Therefore, intensive cessation interventions, such as multifaceted behavioral approaches combined with pharmacotherapy to diminish withdrawal symptoms, may be a potential strategy for this high risk group [44]. More evidence is warranted regarding the development of appropriate cessation interventions for HIV-positive smokers with polysubstance use within the context of limited resources at HIV clinics.

Interestingly, this study found that HIV-positive smokers who were taking Methadone were less motivated to quit smoking. We are unaware of any research on potential impacts of concurrent treatment for illicit drugs and nicotine dependence among PLWHA. However, there is debate on providing interventions on multiple health behaviors simultaneously or sequentially in the general population [47]. The main arguments being that cigarette is a less harmful alternative to illicit drug, and that quitting smoking might result in a negative impact on drug use treatment, mean that smoking cessation interventions are often not provided for smokers in drug addiction treatment [48]. Nevertheless, a meta-analysis suggested that smoking cessation interventions provided during other addiction treatment interventions were associated with a 25% increased likelihood of long-term abstinence from alcohol and illicit drugs, and also effected short-term smoking abstinence [49]. Similarly, results from a randomized trial of 538 stimulant-dependent smokers demonstrated that concurrent smoking cessation and drug use treatment can significantly improve smoking abstinence outcomes without negatively impacting non-nicotine outcomes [50]. Additional research is necessary to address this issue, especially to identify teachable moments among HIV-positive smokers with dual dependence.

The poor understanding of available treatments combined with financial constraints may contribute to our finding that self-help was the most preferred method to quit smoking. This underscores a need to inform smokers about the effectiveness of available smoking cessation treatments. Current U.S. guidelines recommend a combination of counseling and pharmacologic interventions as traditional treatment for general smokers [40]. Smoking cessation efforts targeting PLWHA in Vietnam particularly need to be tailored to meet both the unique characteristics of the HIV-positive population and the specific circumstances in resource-limited settings. Frequent medical visits to monitor disease progress and ART treatment provide good opportunities to integrate smoking cessation treatment into HIV care [44]. Additionally, a study in Vietnam found that brief physician advice was very cost-effective and should be included in the priority list of tobacco control policies [51]. As a first step, pilot research should be conducted to test the feasibility and preliminary efficacy of brief provider-delivered advice to quit smoking at ART clinics in Vietnam. Such an effort would likely require substantial provider training and education efforts. Specifically, recent findings in Vietnam revealed that less than one third of general smokers (29.7%) receive advice from healthcare staff to quit smoking [17].

The strengths of our study include a large sample of HIV-positive smokers in Vietnam. Furthermore, our study settings included both rural and urban areas, and selected clinic sites encompassed ART clinics across all three levels of the health system (i.e. central, provincial, and district clinics). Importantly, validated measures (e.g., FTND, AUDIT-C) used in this study make our findings more reliable and comparable. However, several limitations should also be considered. First, due to the nature of a cross-sectional design, we cannot establish temporal or causal relationships between independent variables and motivation to quit. Second, self-reported data in this study may be compromised by some degree of recall errors and socially desirable responding. Third, the generalization of our study results is limited by the convenience sampling strategy. Fourth, the current study did not collect data on psychosocial factors (e.g., self-efficacy, and perceived risk); thus, other salient smoking-related constructs may underlie the observed findings. Assessing impacts of these factors on motivation to quit is critical to have a better understanding of the mechanism of changing smoking behavior. Finally, motivation to quit was dichotomized in our study instead of using a continuum measure of the Contemplation ladder. Therefore, motivation to quit smoking may not have been measured in full, such as low motivation versus high motivation. Future studies are needed to gain insight into this issue.

## Conclusions

Given the high level of motivation to quit among HIV-positive smokers in Vietnam, smoking cessation should be integrated into HIV care to help smokers achieve successful abstinence, and consequently to reduce adverse health outcomes among PLWHA. Interventions tailored to meet specific needs for individuals with different attitudes on smoking, low income, and polysubstance use may be warranted.

## Abbreviations

ART: Antiretroviral therapy
PLWHA: People living with HIV/AIDS
FTND: Fagerstrom Test for Nicotine Dependence
AUDIT-C: Alcohol Use Disorders Identification Test-Consumption
